# Low total and free triiodothyronine levels are associated with insulin resistance in non-diabetic individuals

**DOI:** 10.1038/s41598-018-29087-1

**Published:** 2018-07-16

**Authors:** Chih-Yuan Wang, Tse-Ya Yu, Shyang-Rong Shih, Kuo-Chin Huang, Tien-Chun Chang

**Affiliations:** 1 Division of Endocrinology and Metabolism, Department of Internal Medicine, National Taiwan University Hospital, Taipei, 100 Taiwan; 2 Department of Internal Medicine, College of Medicine, National Taiwan University, Taipei, 100 Taiwan; 3 Health Management Center, Far-Eastern Memorial Hospital, New Taipei City, 222 Taiwan; 4 Department of Family Medicine, National Taiwan University Hospital, Taipei, 100 Taiwan

## Abstract

This study examined associations of thyroid hormone levels and insulin resistance (IR) in non-diabetic individuals. Using a cross-sectional design, 2007–2008 data from the National Health and Nutrition Examination Survey (NHANES) were analyzed. NHANES participants ≥20 years of age with complete data of interest were included. The homeostatic model assessment (HOMA) was used to quantify IR, and treated as a continuous variable. Self-reported diabetes or a fasting glucose ≥7 mmol/L were used as criteria to exclude diabetic subjects. Race, liver function, obesity, hypertension, dyslipidemia, smoking, physical activity, vigorous recreational activity, 2-hour glucose, hemoglobin A1C (HbA1C), high-density lipoprotein, triglyceride, vitamin D and C-reactive protein were covariates significantly associated with HOMA-IR. A total of 1,560 non-diabetic subjects were included in the analysis. When adjusted for all factors significant in the univariate analysis (race, liver function, obesity, hypertension, dyslipidemia, smoking, physical activity, vigorous recreational activity, 2-hour glucose, HbA1C, high-density lipoprotein, triglyceride, vitamin D, and CRP) low total triiodothyronine (TT3) and low free T3 (FT3) were significantly associated with decreased HOMA-IR (adjusted coefficient = −0.486, 95% confidence interval [CI]: −0.936, −0.036; adjusted coefficient = −1.151, 95% CI: −1.952, −0.350, respectively). Insulin resistance is associated with low thyroid hormone levels in non-diabetic individuals.

## Introduction

Insulin resistance (IR) is defined as a glucose homeostasis disorder involving a decreased sensitivity of muscles, adipose tissue, liver, and other body tissues to insulin, and IR is a hallmark feature of type 2 DM^1^. Thyroid hormones are critically important for regulating energy balance, and the metabolism of glucose and lipids^2^. Thyroid disease is much more common in patients with DM than in the general population, suggesting a possibly relationship between thyroid hormone levels and insulin sensitivity^3^. Thyroid hormones exert both insulin agonistic and antagonistic actions in different organs, and studies have indicated thyroid hormones play a role in the development of IR through complex, intertwined mechanisms of biochemical, genetic, and secretory malfunctions^4–8^. Thyroid dysfunction has also been associated with metabolic syndrome and risk of CVD^9^.

However, some of the associations seem paradoxical. For example, it has been known for decades that hyperthyroidism is associated with IR; however, recently hypothyroidism has been reported to be associated with IR^6^. A recent study that examined data from the Rotterdam Study, a large prospective population-based cohort study, found that low and low-normal thyroid function were risk factors for the development of diabetes, especially in individuals with prediabetes^10^. On the other hand, another recent study found no association between thyroid stimulating hormone (TSH) and free thyroxine (FT4) and IR^11^. A longitudinal study of obese children found that IR and TSH were positively correlated, independent of body status, and a decrease in TSH levels was independently associated with decreases in HOMA-IR^12^. Another study of euthyroid adolescents with risk factors for the development of diabetes reported a correlation between fasting insulin, IR, and serum thyroid hormone levels, but no correlation with serum TSH^13^. Analysis of data from the Tehran Thyroid Study found that low FT4 was independently associated with IR in healthy euthyroid men, but not in women^7^.

Thus, the purpose of this study was to use a large population-based database to examine the associations of thyroid hormone levels and IR in non-diabetic individuals.

## Methods

### Data source

Data from the National Health and Nutrition Examination Survey (NHANES) collected from 2007-2008 were used for this analysis^14^. The NHANES program began in the early 1960s, and has been conducted as a series of surveys focusing on different population groups and health topics. The sample for the NHANES survey is selected to represent the United States population of all ages. Briefly, all of the counties in the United States are divided into 15 groups based on their characteristics. One large county is selected from each group, and together they form the 15 counties that are used for the survey each year. Full details regarding selection down to the household and individual level are available at https://www.cdc.gov/nchs/nhanes/participant.htm.

Health interviews are conducted in participant’s homes. Health measurements are performed in specially-designed and equipped mobile examination centers, which travel to locations throughout the country. The study team consists of a physician, medical and health technicians, and dietary and health interviewers. All participants visit the physician. All participants receive dietary interviews and body measurements. All but very young participants have a blood sample taken and have a dental screening. Depending upon the age of the participant, the rest of the examination includes tests and procedures to assess the various aspects of health. In general, the older the individual, the more extensive the examination. All data are input into computers at the time of collection.

Information from NHANES is made available through an extensive series of publications and articles in scientific and technical journals. For data users and researchers throughout the world, survey data are available on the internet and CD. Further information about background, design, and operation are available on the NHANES https://www.cdc.gov/nchs/nhanes/about_nhanes.htm.

All NHANES surveys receive NCHS Research Ethics Review Board (ERB) Approval (https://www.cdc.gov/nchs/nhanes/irba98.htm). All of the NHANES data are de-identified, and analysis of the data does not require Institutional Review Board approval or informed consent by subjects. The NHANES program can be contacted at: Division of Health and Nutrition Examination Surveys, National Center for Health Statistics, 3311 Toledo Rd, Room 4551, Hyattsville, MD 20782-2064. Or by calling 1-800-232-4636 or by email at https://wwwn.cdc.gov/dcs/ContactUs/Form.

### Study population

This study included NHANES adult participants (≥20 years of age) who were examined at a mobile examination center, who had testing of fasting glucose, insulin, and thyroid hormone levels, and were not missing data of covariates of interest. Pregnant women and individuals with cancer and thyroid disease were excluded.

Subjects with diabetes were also excluded. The presence of DM was based on self-report or the level of fasting glucose. If a patient responded yes when asked if they had ever been told by a doctor or healthcare professional that they had diabetes, and they were taking an oral medication to lower blood sugar, they were classified as having DM. Subjects with a fasting glucose >7 mmol/L, were also defined as having DM and excluded.

### Study variables

#### Insulin resistance

The homeostatic model assessment (HOMA) was used to quantify IR^1^. Using the HOMA method, IR is calculated as HOMA-IR = fasting insulin (μU/mL) × fasting glucose (mmol/L) ÷ 22.5^1^. There is great variability in the definition of IR using the HOMA, and as such in this study HOMA-IR was treated as a continuous variable in the analysis^15,16^.

#### Thyroid function

Thyroid hormones and antibodies measured in the NHANES thyroid profile include TSH, total and free triiodothyronine (TT3, FT3), total and free thyroxine (TT4, FT4), thyroglobulin (TGN), and thyroid peroxidase antibodies (TPOab). Detailed specimen collection, processing instructions, and analytical methods are outlined in the NHANES Laboratory Procedures Manual^14^.

Serum thyroid hormone and antibody levels were classified into three categories: normal (within reference range), low (lower than lower reference range limit), and high (higher than upper reference range limit) based on the reference ranges described in the NHANES Laboratory Procedures Manual. References ranges were: TSH, 0.24–5.4 uIU/mL; TT3, 87–178 ng/dL; FT3, 2.5–3.9 pg/mL; TT4, 6.1–12.2 µg/dL; FT4: 0.6–1.6 ng/dL; TGN: 0–35.0 ng/mL; TPOab: 0–9.0 IU/mL.

#### Demographic data

Age, sex, and race/ethnicity were obtained from the NHANES database. Subjects were grouped by age as follows: 20–40, 41–60, and ≥61 years of age.

#### Comorbidities

Poor liver function was defined as an alanine aminotransferase (ALT) level >40 U/L, or if the subject reported they had been told by a doctor or health professional that they had any kind of liver condition. Obesity was dichotomized, and defined as a body mass index (BMI) ≥30 kg/m^2^.

Chronic kidney disease (CKD) was defined as an estimated glomerular filtration rate (eGFR) <60 ml/min/1.73 m^2^ ^17^. eGFR was calculated based on serum creatinine level using the Modification of Diet in Renal Disease (MDRD) Study equation. Subjects were considered to have cardiovascular disease (CVD) if they answered yes to the question: Have you ever been told you had coronary artery disease, angina pectoris, congestive heart failure, heart attack, or stroke?

The diagnosis of depression was based on Depression Screener (DPQ) questions from the Patient Health Questionnaire, a version of the Prime-MD diagnostic instrument. Questions are based on nine DSM-IV signs and symptoms of depression, and were self-reported by the subjects for the past 2 weeks. The nine questions are scored from “0” (not at all) to “3” (nearly every day). In this study, a total score ≥10 indicated depression.

#### Behavioral characteristics

Current smokers were those who had smoked at least 100 cigarettes during their lifetime, and reported smoking every day or some days. Former smokers were those who reported smoking at least 100 cigarettes during their lifetime, but currently did not smoke. Never smokers were those who reported never having smoked cigarettes.

Alcohol use was based on self-report. Subjects who in their entire life never had at least 12 drinks were defined as never drinkers. Subjects who had at least 12 drinks in their entire life, but had not consumed alcohol in the past 12 months were defined as former drinkers. Subjects who consumed at least 12 drinks in their entire life, and drank on at least 1 day in the past year were considered current drinkers^18^.

The degree of physical activity was based on answers to questions related to daily activities and leisure time activities as outlined in the NHANES Manual. For this study, we calculated the total metabolic equivalent (MET) scores for the activities, as suggested by the NHANES manual^14^. The World Health Organization (WHO) recommendations a physical activity for health cut-off value of 600 MET-min/week. Thus, physical activity was dichotomized as a MET ≥600 or <600.

#### Laboratory studies

Glycated hemoglobin A1C (HbA1C, glycohemoglobin) level was stratified into <5.5%, 5.5–6.4%, and ≥6.5% base on WHO^19^ and other clinical practice guidelines. Serum 25-hydroxy vitamin D (nmol/L) levels were measured at the National Center for Environmental Health, CDC, Atlanta, GA, and were stratified as normal (>75 nmol/L), insufficiency (50–75 nmol/L), and deficiency (<50 nmol/L). C-reactive protein (CRP) was quantitatively determined on a Behring Nephelometer. Detailed specimen collection and processing instructions are discussed in the NHANES Laboratory Procedures Manual^14^.

### Statistical Analysis

Continuous variables were expressed as mean ± standard error; categorical variables were shown as unweighted counts (weighted %). When a sample is weighted in NHANES it is considered to be representative of the U.S. civilian noninstitutionalized population. It is important to utilize the weights in analyses to account for the complex survey design, survey nonresponse, and post-stratification in order to ensure that calculated estimates are truly representative of the U.S. civilian noninstitutionalized population. All analyses included special sample weights [WTSAF2YR (Fasting Subsample 2-year MEC Weight) for 2007–2008], stratum and primary sampling units (PSU) per recommendations from the National Center for Health Statistics (NCHS), and complex sample analysis to address oversampling, non-response, non-coverage, and to provide nationally representative estimates.

Univariate and multivariate linear regression analyses were performed using the Complex Samples General Linear Model (CSGLM) to determine the relationship of the study variables with HOMA-IR. Multivariate linear regression analyses were performed to determine the associations between thyroid hormone levels and HOMA-IR with adjustment of factors found significant in univariate analysis. Factors adjusted for included race, liver function, depression, obesity, hypertension, dyslipidemia, smoking, physical activity, vigorous recreational activity, moderate recreational activity, 2-hour glucose, HbA1C, high-density lipoprotein, triglyceride, vitamin D, and CRP.

All statistical assessments were 2-sided, and evaluated at the 0.05 level of significance. Statistical analyses were performed using IBM SPSS statistical software version 22 for Windows (IBM Corp., Armonk, New York, USA).

## Result

### Sample and participant characteristics

**Figure 1:**
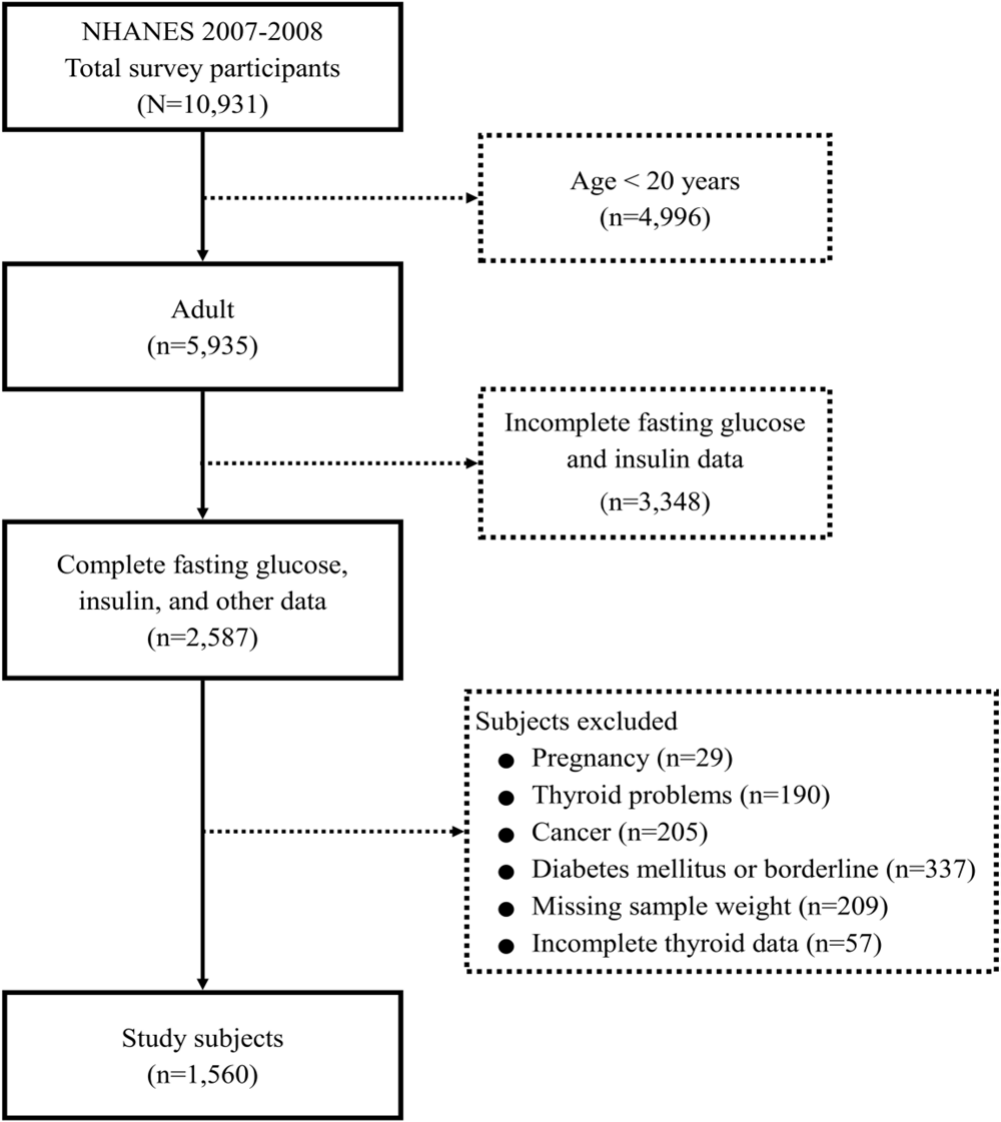
Flow diagram of subject inclusion.

A total of 10,931 participants were included in the NHANES 2007-2008 cycle. Of these 2,587 were 20 years of age or older and had complete data of interest. After excluding participants who did not meet the inclusion criteria (Fig. 1), 1,560 individuals were included in the present analysis. In NHANES, a sample weight is assigned to each sample person. When a sample is weighted, it becomes representative of the U.S. Census civilian noninstitutionalized population. Using the NHANES sample weight, the analytic sample size of 1,560 was equivalent to a population-based sample size of 155,402,992 individuals.

Most of participants were between 20 and 40 years of age (47.3%), were male (51.1%), and were non-Hispanic White (68.9%). Other notable characteristics included never having smoked (56.0%), current alcohol consumption (66.0%), metabolic equivalent of task ≥600 (69.6%), glycohemoglobin <5.5% (61.6%), and vitamin D insufficiency (37.4%) (Table 1). The mean of HOMA-IR was 2.71 ± 0.08.

**Table 1:** Subject characteristics (unweighted N = 1,560; weighted N = 155,402,992).

Characteristics	Total (N = 1560)
**HOMA-IR**	2.71 ± 0.08
**Demographic data**	
Age	
20–40 years	667 (47.3)
41–60 years	529 (38.7)
≥61 years	364 (14.0)
Sex	
Female	764 (48.9)
Male	796 (51.1)
Race	
Non-Hispanic white	723 (68.9)
Non-Hispanic black	288 (10.7)
Mexican American	283 (8.6)
Other Hispanic	195 (5.2)
Other race	71 (6.6)
**Comorbidities**	
Liver function	
Normal	1389 (89.7)
Poor	170 (10.3)
Chronic kidney disease	
No, eGFR ≥60 (mL/min/1.73 m^2^)	1462 (94.9)
Yes, eGFR <60 (mL/min/1.73 m^2^)	98 (5.1)
Cardiovascular disease	
No	1446 (94.9)
Yes	114 (5.1)
Depression	
No	1355 (89.2)
Yes	111 (5.5)
Obesity	
No	1067 (71.4)
Yes	475 (27.7)
Hypertension	
No	1137 (75.0)
Yes	421 (24.9)
Dyslipidemia	
No	619 (39.8)
Yes	940 (60.0)
**Health behavior**	
Smoking	
Never	857 (56.0)
Former	353 (22.1)
Current	349 (21.9)
Alcohol consumption	
Never	176 (9.3)
Former drinker	207 (11.9)
Current drinker	944 (66.0)
Physical activity	
Metabolic equivalent of task ≥600	1010 (69.6)
Metabolic equivalent of task <600	548 (30.4)
Vigorous recreational activity	
No	1169 (69.2)
Yes	391 (30.8)
Moderate recreational activity	
No	965 (57.1)
Yes	595 (42.9)
**Laboratory studies**	
Two Hour Glucose (mmol/L)	6.17 ± 0.08
Glycohemoglobin (%)	
<5.5	847 (61.6)
5.5–6.4	695 (37.9)
≥6.5	15 (0.3)
High-density lipoprotein (mmol/L)	1.39 ± 0.02
Triglyceride (mmol/L)	1.45 ± 0.03
Serum vitamin D	
Normal (>75 nmol/L)	298 (24.7)
Insufficiency (50–75 nmol/L)	559 (37.4)
Deficiency (<50 nmol/L)	518 (25.8)
C-reactive protein (mg/dL)	0.31 ± 0.01

eGFR, estimated glomerular filtration rate.

Continuous variables were shown mean ± standard error; categorical variables were shown unweighted counts (weighted %). Numbers may not add to full sample due to missing data.

### Analyses of associated factors on HOMA-IR

Univariate linear regression analysis indicated that race, liver function, obesity, hypertension, dyslipidemia, smoking, physical activity, vigorous recreational activity, 2-hour glucose, HbA1C, high-density lipoprotein, triglyceride, vitamin D, and CRP were significantly associated with HOMA-IR (Supplement Table 1). In addition, low TT3 (coefficient = −0.747, 95% CI: −1.199, −0.294), and low FT4 (coefficient = 1.925, 95% CI: 0.233, 3.617) were associated with HOMA-IR (Table 2).

**Table 2:** Relationship between thyroid hormone levels and insulin resistance.

Thyroid hormone	Total (N = 1560)	Univariate	Multivariate
		Coefficient (95% CI)	Coefficient (95% CI)
Thyroid stimulating hormone (TSH) (mIU/L)	2.01 ± 0.08		
Normal (0.24–5.4 mIU/L)	1506 (97.0)	Reference	Reference
Low (<0.24 mIU/L)	16 (0.8)	−0.632 (−1.291, 0.027)	−0.564 (−1.288, 0.161)
High (>5.4 mIU/L)	38 (2.2)	0.228 (−0.606, 1.061)	0.204 (−0.418, 0.827)
Total triiodothyronine (TT3) (ng/dL)	115.69 ± 1.01		
Normal (87–178 ng/dL)	1445 (93.0)	Reference	Reference
Low (<87 ng/dL)	97 (6.1)	**−0.747 (−1.199, −0.294)**	**−0.486 (−0.936, −0.036)**
High (>178 ng/dL)	18 (0.9)	0.661 (−0.321, 1.643)	0.586 (−0.211, 1.382)
Free triiodothyronine (FT3) (pg/mL)	3.27 ± 0.02		
Normal (2.5–3.9 pg/mL)	1470 (94.3)	Reference	Reference
Low (<2.5 pg/mL)	15 (0.7)	−0.647 (−1.493, 0.198)	**−1.151 (−1.952, −0.35)**
High (>3.9 pg/mL)	75 (5.0)	0.206 (−0.088, 0.501)	−0.092 (−0.449, 0.265)
Total thyroxine (TT4) (ug/dL)	7.69 ± 0.06		
Normal (6.1–12.2 ug/dL)	1372 (86.6)	Reference	Reference
Low (<6.1 ug/dL)	174 (12.7)	−0.141 (−0.553, 0.270)	−0.054 (−0.347, 0.240)
High (>12.2 ug/dL)	14 (0.7)	0.056 (−0.693, 0.806)	−0.214 (−0.632, 0.203)
Free thyroxine (FT4) (ng/dL)	0.78 ± 0.01		
Normal (0.6–1.6 ng/dL)	1532 (97.9)	Reference	Reference
Low (<0.6 ng/dL)	25 (2.0)	**1.925 (0.233, 3.617)**	1.372 (−0.282, 3.025)
High (>1.6 ng/dL)	3 (0.1)	−0.606 (−1.389, 0.178)	−1.857 (−4.102, 0.387)
Thyroglobulin (ng/mL)	15.49 ± 0.95		
Normal (0–35.0 ng/mL)	1446 (93.5)	Reference	Reference
High (>35.0 ng/mL)	114 (6.5)	0.715 (−0.062, 1.492)	0.523 (−0.373, 1.418)
Thyroid peroxidase antibodies (IU/mL)	16.62 ± 1.85		
Normal (0–9.0 IU/mL)	1422 (90.3)	Reference	Reference
High (>9.0 IU/mL)	138 (9.7)	−0.022 (−0.472, 0.429)	0.008 (−0.266, 0.282)

CI, confidence interval.

Bold indicates significant factor, p < 0.05.

Thyroid hormone levels were shown mean ± standard error; thyroid hormone group numbers were shown unweighted counts (weighted %).

Multivariate model: Adjusted for thyroid hormones, race, liver function, depression, obesity, hypertension, dyslipidemia, smoking, physical activity, vigorous recreational activity, moderate recreational activity, 2-hour glucose, glycohemoglobin, high-density lipoprotein, triglyceride, vitamin D, and C-reactive protein.

Linear regression analysis revealed varying degrees of associations with HOMA-IR (Model 1, Model 2, and Model 3 in Supplemental Table 2). When adjusted for HbA1C level, low TT3 was significantly associated with decreased HOMA-IR (adjusted coefficient = −0.745, 95% CI = −1.21, −0.281). On the other hand, high FT3 and low FT4 were significantly associated with increased HOMA-IR (adjusted coefficient = 0.370, 95% CI = 0.073, 0.668; adjusted coefficient = 1.776, 95% CI = 0.181, 3.372, respectively). After further adjusting for obesity, decreased HOMA-IR is significantly associated with low TSH, low TT3, and low FT3 (adjusted coefficient = −0.663, 95% CI: −1.170, −0.156; adjusted coefficient = −0.470, 95% CI: −0.903, −0.037; adjusted coefficient = −0.784, 95% CI: −1.444, −0.124, respectively). However, HOMA-IR is increased in low FT4 (adjusted coefficient = 1.656, 95% CI: 0.211, 3.101). No association was found between high FT3 and HOMA-IR (adjusted coefficient = 0.110, 95% CI: −0.305, 0.525). With additional adjustments for liver function, decreased HOMA-IR was significantly associated with low TSH, low TT3, and low FT3 (adjusted coefficient = −0.593, 95% CI: −1.091, −0.095; adjusted coefficient = −0.437, 95% CI: −0.859, −0.015; adjusted coefficient = −0.821, 95% CI: −1.399, −0.242, respectively). However, after adjustment for liver function, HOMA-IR was not associated with low FT4 (adjusted coefficient = 1.514, 95% CI = −0.001, 3.030).

When adjusted for all factors found to be significant in the univariate analysis (race, liver function, obesity, hypertension, dyslipidemia, smoking, physical activity, vigorous recreational activity, 2-hour glucose, HbA1C, high-density lipoprotein, triglyceride, vitamin D, and CRP) low TT3 and low FT3 were significantly associated with decreased HOMA-IR (adjusted coefficient = −0.486, 95% CI: −0.936, −0.036; adjusted coefficient = −1.151, 95% CI: −1.952, −0.350, respectively) (Table 2).

## Discussion

This population-based study sought to examine the associations of thyroid hormone levels and IR, using HOMA-IR as a measure of IR, in non-diabetic individuals. The results showed that HOMA-IR was associated with low FT3 and low TT3.

The interplay between thyroid hormones, β cell function, liver gluconeogenesis and glycogenolysis, intestinal absorption of glucose, and metabolism of lipids is complex and only just beginning to be understood^4,6,9^. One of the more interesting observations is that both hypo- and hyperthyroidism have been found to be associated with IR^4,20^. While the pathogenic mechanisms of this finding has yet to be fully described, Kapadia *et al*.^4^ reported that it was related to altered glucose and lipid metabolism that occurs in both hypo- and hyperthyroidism. On the other hand, evidence suggests that in hyperthyroidism impaired glucose tolerance is primarily the result of hepatic IR, whereas in the case of hypothyroidism IR of peripheral tissue is predominant^21^.

A study of Hispanic persons with no history of thyroid disease or DM found that low thyroid function, even measures at the low end of the normal reference range, were associated with higher cholesterol, glucose, insulin, and HOMA-IR levels^22^. Other study has shown that low FT4 was independently associated with IR in healthy euthyroid males. Lambadiari *et al*.^5^ studied healthy control subjects, first-degree relatives of individuals with type 2 DM, subjects with impaired glucose tolerance, and subjects with type 2 DM and found that FT4 and FT3 levels that were increased, yet still within the normal range were positively associated with IR. Aksoy *et al*.^23^ interestingly found a significant positive correlation between BMI and HOMA-IR in persons with high TSH, and those with subclinical hypothyroidism.

While the current study only included adults, other studies have found associations between thyroid function and IR in adolescents. For example, Garduño-Garcia *et al*.^13^ reported a correlation between fasting insulin, HOMA-IR, and thyroid hormone levels in euthyroid adolescents with risk factors for the development of DM. With respect to other variables examined in this study, sedentary lifestyle has been shown to be associated with IR^24^. Analysis of longitudinal data by Peixoto de Miranda *et al*.^25^ found TSH was not associated with CRP; however, the authors indicated IR and obesity were important confounders. Young prediabetic individuals have been found to have greater impairment of insulin secretion than older prediabetic individuals^26^. The presence of advanced glycation end-products and nitric oxide has also been show to play a role in mediating IR and obesity^27,28^.

The primary strength of this study is that the data are representative of the population of the United States. However, there are limitations of this study that need to be taken into consideration. These limitations are those that limit all population-based analysis. Data was collected from a single visit, and longitudinal follow-up was not available. While correlations can be determined, the data precludes the determination of cause and effect. The study was limited to data from the NHANES 2007–2008 cycle. Although there are also data of thyroid hormone levels in the 2009–2012 database, the thyroid data in these years are subsamples of the NHANES sample, and NHANES strongly advises not to combine different subsamples from a single survey cycle in any analysis. We did not separately examine males and females, and study has shown there are differences between males and females with respect to factors associated with IR^29,30^.

## Conclusions

In conclusion, HOMA-IR was associated with low FT3 and low TT3 levels in non-diabetic individuals. These findings add further data to the study of the association between thyroid function and the development of diabetes.

## Electronic supplementary material

Supplementary Tables
