# Supplementary material for: Low total and free triiodothyronine levels are associated with insulin resistance in non-diabetic individuals
Source: Sci Rep. 2018 Jul 16;8:10685. doi: 10.1038/s41598-018-29087-1 (PMC6048097; doi:10.1038/s41598-018-29087-1)
Supplement: Supplementary file 1 — Supplementary Tables [file 41598_2018_29087_MOESM1_ESM.pdf]

Low total and free triiodothyronine levels are associated with insulin resistance in non-diabetic individuals

Short title: Thyroid hormones and insulin resistance

Chih-Yuan Wang[1,2,*], Tse-Ya Yu[3], Shyang-Rong Shih[1,2], Kuo-Chin Huang[4], Tien-Chun Chang[1,2]

[1] Division of Endocrinology and Metabolism, Department of Internal Medicine, National Taiwan University Hospital, Taipei 100, Taiwan

[2] Department of Internal Medicine, College of Medicine, National Taiwan University, Taipei 100, Taiwan

[3] Health Management Center, Far-Eastern Memorial Hospital, New Taipei City 222, Taiwan

[4] Department of Family Medicine, National Taiwan University Hospital, Taipei 100, Taiwan

*Corresponding author: Chih-Yuan Wang, MD, Ph.D

Division of Endocrinology and Metabolism, Department of Internal Medicine, National Taiwan University Hospital and Department of Internal Medicine, College of Medicine, National Taiwan University, Taipei 100, Taiwan

TEL: +886-2-23123456 ext 63864

FAX: +886-2-23934176

Email: cyw1965@gmail.com

Supplemental Table 1. Relations between subject characteristics and insulin resistance

Variables	Univariate
	Coefficient (95% CI)
**Demographic data**	
Age	
20-40 years	Reference
41-60 years	0.163 (-0.164, 0.490)
≥ 61 years	0.143 (-0.123, 0.410)
Sex	
Female	Reference
Male	0.240 (-0.042, 0.522)
Race	
Non-Hispanic white	Reference
Non-Hispanic black	0.061 (-0.394, 0.517)
Mexican American	**0.818 (0.467, 1.169)**
Other Hispanic	0.533 (-0.243, 1.308)
Other race	0.107 (-0.494, 0.708)
**Comorbidities**	
Liver function	
Normal	Reference
Poor	**1.320 (0.854, 1.785)**
Chronic kidney disease	
No, eGFR ⩾ 60 (mL/min/1·73m^2)	Reference
Yes, eGFR < 60 (mL/min/1·73m^2)	-0.222 (-0.546, 0.102)
Cardiovascular disease	
No	Reference
Yes	0.403 (-0.011, 0.816)
Depression	
No	Reference
Yes	0.594 (-0.179, 1.367)
Obesity	
No	Reference
Yes	**2.076 (1.750, 2.403)**
Hypertension	
No	Reference
Yes	**1.020 (0.670, 1.370)**
Dyslipidemia	
No	Reference
Yes	**0.738 (0.454, 1.022)**
**Health behavior**	

Smoking	
Never	Reference
Former	0.050 (-0.174, 0.275)
Current	**-0.278 (-0.550, -0.005)**
Alcohol consumption	
Never	Reference
Former drinker	0.297 (-0.227, 0.821)
Current drinker	0.053 (-0.224, 0.330)
Physical activity	
Metabolic equivalent of task ≥ 600	Reference
Metabolic equivalent of task < 600	**0.590 (0.237, 0.942)**
Vigorous recreational activity	
No	Reference
Yes	**-0.622 (-0.850, -0.394)**
Moderate recreational activity	
No	Reference
Yes	-0.339 (-0.697, 0.018)
**Laboratory studies**	
Two Hour Glucose (mmol/L)	**0.290 (0.224, 0.357)**
Glycohemoglobin (%)	
< 5.5	Reference
5.5-6.4	**0.807 (0.507, 1.107)**
≥ 6.5	0.762 (-0.324, 1.847)
High-density lipoprotein (mmol/L)	**-1.790 (-2.280, -1.301)**
Triglyceride (mmol/L)	**0.481 (0.261, 0.701)**
Serum vitamin D	
Normal (> 75 nmol/L)	Reference
Insufficiency (50 – 75 nmol/L)	**0.689 (0.203, 1.175)**
Deficiency (< 50 nmol/L)	**1.000 (0.442, 1.558)**
C-reactive protein (mg/dL)	**1.016 (0.507, 1.525)**

CI, confidence interval; eGFR, estimated glomerular filtration rate.
Bold indicates significant factor, $p < 0.05$.

Supplemental Table 2. Relations between thyroid hormone levels and insulin resistance adjusting for different factors.

	Model 1	Model 2	Model 3
	Coefficient (95% CI)	Coefficient (95% CI)	Coefficient (95% CI)
**Thyroid stimulating hormone (TSH) (mIU/L)**			
Normal (0.24-5.4 mIU/L)	Reference	Reference	Reference
Low (< 0·24 mIU/L)	-0.542 (-1.191, 0.107)	**-0.663 (-1.170, -0.156)**	**-0.593 (-1.091, -0.095)**
High (> 5·4 mIU/L)	0.159 (-0.598, 0.916)	0.306 (-0.361, 0.973)	0.376 (-0.288, 1.040)
**Total triiodothyronine (TT3) (ng/dL)**			
Normal (87-178 ng/dL)	Reference	Reference	Reference
Low (< 87 ng/dL)	**-0.745 (-1.210, -0.281)**	**-0.470 (-0.903, -0.037)**	**-0.437 (-0.859, -0.015)**
High (> 178 ng/dL)	0.756 (-0.070, 1.582)	0.363 (-0.373, 1.099)	0.415 (-0.372, 1.202)
**Free triiodothyronine (FT3) (pg/mL)**			
Normal (2.5-3.9 pg/mL)	Reference	Reference	Reference
Low (< 2.5 pg/mL)	-0.837 (-1.678, 0.003)	**-0.784 (-1.444, -0.124)**	**-0.821 (-1.399, -0.242)**
High (> 3.9 pg/mL)	**0.370 (0.073, 0.668)**	0.110 (-0.305, 0.525)	-0.006 (-0.454, 0.443)
**Total thyroxine (TT4) (ug/dL)**			
Normal (6.1-12.2 ug/dL)	Reference	Reference	Reference
Low (< 6.1 ug/dL)	-0.097 (-0.518, 0.324)	-0.081 (-0.410, 0.248)	-0.115 (-0.470, 0.240)
High (> 12.2 ug/dL)	-0.035 (-0.642, 0.572)	-0.045 (-0.479, 0.388)	0.048 (-0.379, 0.474)
**Free thyroxine (FT4) (ng/dL)**			
Normal (0.6-1.6 ng/dL)	Reference	Reference	Reference
Low (< 0.6 ng/dL)	**1.776 (0.181, 3.372)**	**1.656 (0.211, 3.101)**	1.514 (-0.001, 3.030)
High (> 1.6 ng/dL)	-0.718 (-1.963, 0.528)	-0.131 (-1.269, 1.007)	-0.066 (-1.222, 1.090)
**Thyroglobulin (ng/mL)**			
Normal (0-35.0 ng/mL)	Reference	Reference	Reference
High (> 35.0 ng/mL)	0.639 (-0.064, 1.342)	0.441 (-0.302, 1.185)	0.497 (-0.256, 1.251)
**Thyroid peroxidase antibody (IU/mL)**			
Normal (0-9.0 IU/mL)	Reference	Reference	Reference
High (> 9.0 IU/mL)	-0.050 (-0.462, 0.361)	-0.014 (-0.380, 0.352)	-0.039 (-0.391, 0.313)

CI, confidence interval.
Model 1: Adjusted for thyroid hormone and glycohemoglobin.
Model 2: Adjusted for thyroid hormone, glycohemoglobin, and obesity.
Model 3: Adjusted for thyroid hormone, glycohemoglobin, obesity and liver function.
Bold indicates significant factor, $p < 0.05$.
